# Erythropoietin exposure of isolated pancreatic islets accelerates their revascularization after transplantation

**DOI:** 10.1007/s00592-021-01760-4

**Published:** 2021-07-12

**Authors:** Maximilian M. Menger, Lisa Nalbach, Leticia P. Roma, Matthias W. Laschke, Michael D. Menger, Emmanuel Ampofo

**Affiliations:** 1 Institute for Clinical and Experimental Surgery, Saarland University, 66421 Homburg, Saar Germany; 2 Department of Trauma and Reconstructive Surgery, Faculty of Medicine, BG Hospital Tuebingen, Eberhard-Karls-University Tuebingen, Tuebingen, Germany; 3 Biophysics Department, Center for Human and Molecular Biology, Saarland University, 66421 Homburg, Saar Germany

**Keywords:** Erythropoietin, Islets, Transplantation, Revascularization, Diabetes, Angiogenesis

## Abstract

**Aims:**

The exposure of isolated pancreatic islets to pro-angiogenic factors prior to their transplantation represents a promising strategy to accelerate the revascularization of the grafts. It has been shown that erythropoietin (EPO), a glycoprotein regulating erythropoiesis, also induces angiogenesis. Therefore, we hypothesized that EPO exposure of isolated islets improves their posttransplant revascularization.

**Methods:**

Flow cytometric, immunohistochemical and quantitative real-time (qRT)-PCR analyses were performed to study the effect of EPO on the viability, cellular composition and gene expression of isolated islets. Moreover, islets expressing a mitochondrial or cytosolic H_2_O_2_ sensor were used to determine reactive oxygen species (ROS) levels. The dorsal skinfold chamber model in combination with intravital fluorescence microscopy was used to analyze the revascularization of transplanted islets.

**Results:**

We found that the exposure of isolated islets to EPO (3 units/mL) for 24 h does not affect the viability and the production of ROS when compared to vehicle-treated and freshly isolated islets. However, the exposure of islets to EPO increased the number of CD31-positive cells and enhanced the gene expression of insulin and vascular endothelial growth factor (VEGF)-A. The revascularization of the EPO-cultivated islets was accelerated within the initial phase after transplantation when compared to both controls.

**Conclusion:**

These findings indicate that the exposure of isolated islets to EPO may be a promising approach to improve clinical islet transplantation.

## Introduction

Pancreatic islet transplantation is a promising therapeutic strategy for the reestablishment of glucose homeostasis in patients suffering from type 1 diabetes [1]. This minimally invasive approach has the advantage to reduce the surgical trauma associated with whole organ pancreas transplantation and to prevent complications induced by the exocrine pancreatic tissue. However, insufficient graft revascularization leads to apoptotic and necrotic islet cell death during the initial posttransplant period and, thus, represents a major drawback of islet transplantation [2]. Accordingly, the improvement of graft revascularization is of major clinical interest.

Several studies have reported that the glycoprotein erythropoietin (EPO) is capable of inducing the formation of new blood vessels [3, 4]. This is mediated by the binding of EPO to its corresponding receptor (EPOR) on endothelial cells, which activates pro-angiogenic signaling pathways [5, 6]. Pancreatic β-cells also express EPOR and the cultivation of islets with EPO protects β-cells from cytokine-induced apoptosis [7]. Moreover, in vivo injection of EPO reduces β-cell damage by promoting anti-oxidative processes [8, 9]. We have previously shown that pretreatment of mice with EPO significantly accelerates the revascularization of transplanted islets [10]. However, an adequate pretreatment of patients with EPO is hard to achieve under clinical conditions, because there is little time between receiving the appropriate donor pancreas and the islet transplantation itself. Hence, the treatment of the isolated islets with EPO prior to transplantation may be an alternative approach to improve their revascularization.

To test this hypothesis, we cultivated mouse isolated islets with EPO (CI + EPO) or vehicle (CI + vehicle) for 24 h and assessed their viability, reactive oxygen species (ROS) levels, cellular composition and gene expression. Moreover, we used freshly isolated mouse islets (FI) as an additional control. In vivo, CI + EPO, CI + vehicle and FI were transplanted into dorsal skinfold chambers of syngeneic recipient mice to analyze the islets’ revascularization by means of intravital fluorescence microscopy and immunohistochemistry. Here, we show for the first time that EPO cultivation accelerates the revascularization of transplanted islets during the initial post-transplantation phase.

## Material and methods

### Chemical and biological reagents

Collagenase NB 4G was purchased from SERVA Elektrophoresis GmbH (Heidelberg, Germany). Neutral red, fluorescein isothiocyanate (FITC)-dextran 150,000, rhodamine 6G and Hoechst 33,342 were purchased from Sigma-Aldrich (Taufkirchen, Germany). Mayer’s hemalaun was purchased from Merck (Darmstadt, Germany), ketamine (Ursotamin®) from Serumwerke Bernburg (Bernburg, Germany) and xylazine (Rompun®) from Bayer (Leverkusen, Germany). HepatoQuick® and EPO-β (recombinant human EPO, NeoRecormon®) were purchased from Roche (Basel, Switzerland). The antibody anti-EPOR (BS1424R) was purchased from Fisher Scientific (Hampton, USA). The antibody anti-CD31 (DIA310) was purchased from Dianova (Germany). The antibody anti-green fluorescent protein (GFP) was purchased from Rockland Immunochemical Inc. (Limerick, USA) and the antibodies anti-insulin as well as 3-amino-9-ethylcarbazole (AEC Substrate System) from Abcam (Cambridge, UK).

### Animals

Twelve- to 24-week-old transgenic FVB/N-TgN (Tie2/GFP) 287 Sato mice (Institute for Clinical & Experimental Surgery, Homburg/Saar, Germany) with a body weight of 25–30 g were used as donors for islet isolation. These mice express the reporter gene GFP under the transcriptional control of the endothelial Tie2 promotor [11]. Eight to 10-week-old FVB/N wild-type mice with a body weight of 22–27 g served as recipients for islet transplantation. Transgenic C57BL/6 mice expressing the H_2_O_2_ sensor roGFP2-Orp1 in the cytosol (roGFP2-Orp1) or in the mitochondria (mito-roGFP2-Orp1) were used for intra-islet redox measurements. This mice have been previously reported in detail [12]. All experiments were performed according to the German legislation on protection of animals and the National Institutes of Health (NIH) Guide for the Care and Use of Laboratory Animals (Institute of Laboratory Animal Resources, National Research Council, Washington DC, USA). The experiments were approved by the local governmental animal protection committee (permission number: 58/2015).

### Isolation and cultivation of pancreatic islets

Mice were anesthetized by intraperitoneal (i.p.) injection of ketamine (80 mg/kg body weight) and xylazine (12 mg/kg body weight). Following cervical dislocation and midline laparotomy, the pancreatic duct was injected with 1 mg/mL collagenase NB 4G containing 25 µL/mL neutral red solution and pancreatic islets were isolated as described previously in detail [13]. Isolated islets were then cultivated in Dulbecco's Modified Eagle's Medium (DMEM) containing 3 units/mL EPO (CI + EPO) or equivalent volume of saline (CI + vehicle) for 24 h at 37 °C and 5% CO_2_.

### NAD(P)H measurement

Twenty-five islets per well of a 96-well plate were exposed 3 units/mL EPO (CI + EPO) or equivalent volume of saline (CI + vehicle) for 24 h. Thereafter, the NAD(P)H levels of CI + EPO, CI + vehicle and FI were determined, using the CLARIOstar Microplate Reader (BMG LABTECH, Ortenberg, Germany). NAD(P)H was detected in islets after excitation at 340 nm.

### Quantitative real time (qRT)-PCR

Total RNA was isolated by QIAzol lysis reagent and cDNA was synthesized from 1 μg of total RNA according to the manufacturer’s instructions of QuantiNova Reverse Transcription Kit (Qiagen). ORA qPCR Green ROX L Mix (highQu) was used for qRT-PCR. β-Actin served as internal control for mRNA detection. The data analysis was performed by the MiniOpticon Real-Time PCR System (Bio-Rad).

Forward and reverse primers were used at a concentration of 700 nM solved in RNase/DNase-free H_2_O. Primer sequences for qPCR were as follows: β-Actin forward 5′- CCTAGGCACCAGGGTGTGAT-3′, reverse 5′-TCTCCATGTCGTCCCAGTTG-3′; Insulin forward 5′-GGGGAGCGTGGCTTCTTCTA-3′, reverse 5′-GGGGACAGAATTCAGTGGCA-3′; and VEGF-A forward 5′- GCTGTACCTCCACCATGCCAAG-3′, reverse 5’-CGCACTCCAGGGCTTCATCG-3′.

### Neutral red and trypan blue staining

CI + EPO, CI + vehicle and FI were washed in PBS and incubated for 2 min at room temperature with neutral red (1:100) or trypan blue (1:100). Subsequently, the islets were washed again with phosphate buffer saline (PBS) and cellular stainings were visualized by bright field images using a 20 × objective of a BX60 microscope (Olympus, Hamburg, Germany).

### Dorsal skinfold chamber

The revascularization of transplanted islets was analyzed by using the dorsal skinfold chamber model. The chamber was prepared and implanted as described previously in detail [14]. Briefly, mice were anesthetized by i.p. injection of ketamine (80 mg/kg body weight) and xylazine (12 mg/kg body weight). Thereafter, two symmetrical titanium frames were implanted on the extended dorsal skinfold of the animals. In a circular area of 15 mm, skin, subcutis, striated muscle and both retractor muscles were completely removed. The remaining layers, consisting of striated muscle, subcutis and skin, were sealed by a removable cover glass, providing direct microscopic access to the microcirculation of the chamber. After the procedure, the tissue was allowed to recover from surgical trauma for 72 h.

For the transplantation of pancreatic islets, mice were anesthetized, the cover glass was removed and the chamber was washed twice with saline. Then, 6 to 8 isolated islets were transplanted onto the striated muscle tissue. After transplantation, the chamber was sealed again with a new cover glass.

### Intravital fluorescence microscopy

A plexiglas stage was used for the fixation of the anesthetized mice. The animals received a retrobulbary, intravenous injection of 0.05 mL 5% FITC-dextran for contrast enhancement of blood plasma. Moreover, 0.05 mL 2% rhodamine 6G, which accumulates in endocrine but not in striated muscle tissue by extravasation from fenestrated endothelium, was intravenously given for the visualization of endocrine tissue revascularization [15]. Then, the dorsal skinfold chamber was positioned under a Zeiss microscope (Zeiss, Oberkochen, Germany) with a 100 W mercury lamp attached to a blue (excitation wavelength: 450–490 nm/emission wavelength: > 515 nm) and a green (530–560 nm/ > 585 nm) filter block. The microscopic images were recorded for off-line evaluation.

Quantitative analysis of the microscopic images was performed by the computer-assisted image analysis system CapImage (Zeintl, Heidelberg, Germany) and included the determination of the grafts’ initial size (mm^2^), revascularized area (mm^2^), functional capillary density (cm/cm^2^) and endocrine revascularization as previously described [16, 17]. We further measured microhemodynamic parameters, i.e., the diameter (µm) and centerline red blood cell (RBC) velocity (µm/s), of individual microvessels within the grafts [16, 17]. Moreover, we assessed the take rate in % (the fraction of engrafted islets on day 14 in relation to the number of transplanted islets).

### Experimental protocol

Pancreatic islets were isolated from GFP-positive FVB/N donor mice. A total number of 18 FVB/N wild-type mice were equipped with dorsal skinfold chambers. The mice were randomly assigned to three experimental groups of six animals each. CI + EPO, CI + vehicle as well as FI were transplanted into dorsal skinfold chambers of recipient mice. Repetitive intravital fluorescence microscopy was performed on days 0, 3, 6, 10 and 14 after islet transplantation. Subsequently, the islet-containing chamber tissue was excised for further histological and immunohistochemical analyses.

### Immunohistochemistry

For the ex vivo experiments, islets from three mice were isolated and pooled. Five islets were transferred into a well of a 96-well plate and exposed to vehicle (CI + vehicle) or EPO (CI + EPO) for 24 h (*n* = 9 wells each). Thereafter, CI + vehicle, CI + EPO and FI were embedded in a mixture of 100 µL HepatoQuick®, 50 µL human citrate plasma and 10 µL of 10% CaCl_2_. The resulting clots were fixed for 24 h in 4% formalin. The formalin-fixed specimens were then embedded in paraffin and 3-μm-thick sections were cut. The sections were stained with an anti-insulin antibody (1:100), an anti-CD31 antibody (1:200) as well as Hoechst 33342 to detect the cell nuclei. The number of insulin and CD31-positive cells in % of all islet cells (5 islets per clot) were analyzed by means of fluorescence microscopy (BX60F; Olympus, Hamburg, Germany). To detect EPOR, the sections were stained with hematoxylin and an anti-EPOR antibody (1:200).

For the analyses of transplanted islets, we generated immunohistological sections from 5 mice containing at least 3 transplanted islets per section. The sections were stained with an anti-insulin antibody (1:100), an anti-CD31 antibody (1:200), an anti-GFP antibody (1:200) as well as Hoechst 33342 to detect the cell nuclei. The number of insulin- and CD31-positive cells in % of all islet cells and the number of GFP/CD31-positive cells in % of all CD31-positive cells from three sections per mouse were analyzed by means of fluorescence microscopy (BX60F; Olympus, Hamburg, Germany).

### Data and statistical analysis

After testing the data for normal distribution and equal variance, differences between multiple groups were assessed by one-way ANOVA followed by the Tukey post hoc test including the correction of the α-error according to Bonferroni probabilities. Statistics were performed by GraphPad Prism (version 8). All values are expressed as scatter plots with median and 95% confidence interval. Statistical significance was accepted for *P* < 0.05.

## Results

### Effect of EPO cultivation on isolated islet viability

**Fig. 1:**
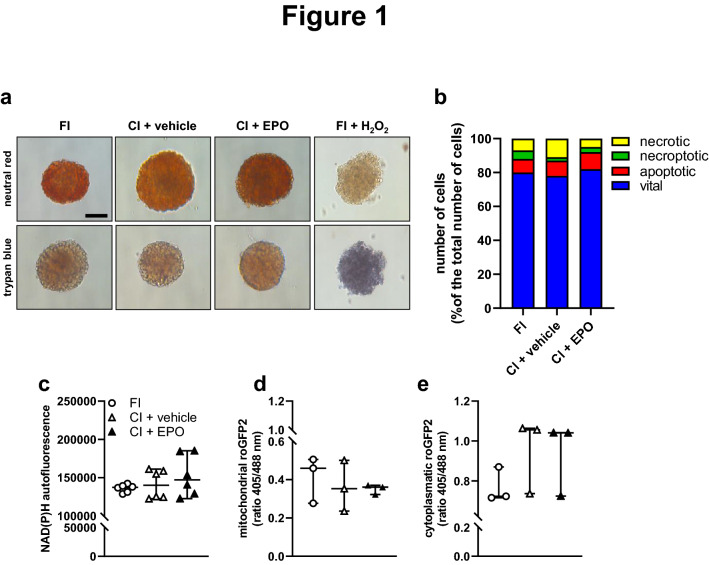
Effect of EPO cultivation on islet viability. **a** Neutral red and trypan blue stainings of freshly isolated islets (FI), vehicle-cultivated islets (CI + vehicle) and EPO-cultivated islets (CI + EPO). FI incubated for 24 h with 0.2% H_2_O_2_ served as control. Scale bar: 70 µm. **b** Quantitative analysis of propidium iodide / annexin V-stained cells from FI, CI + vehicle and CI + EPO subdivided in necrotic, necroptotic, apoptotic and vital cells in % of the total number of cells (n = 6 each). **c** Quantitative analysis of the NAD(P)H levels within FI, CI + vehicle, and CI + EPO (n = 6 each). Data are shown as scatter blots with median and 95% confidence interval. **d** and **e** Quantitative analysis of the ratio of the mitochondrial ROS sensor **e** and the cytosolic ROS sensor **f** (*n* = 3 each). Data are shown as scatter blots with median and 95% confidence interval

In a first set of experiments, we performed neutral red- and trypan blue stainings to assess the effect of 24 h EPO cultivation on the viability of isolated islets. We did not detect any differences between FI, CI + vehicle and CI + EPO (Fig. 1a). We additionally performed flow cytometric analyses of Annexin V / propidium iodide-stained cells and NAD(P)H measurements (Fig. 1b and c). The results of these detailed viability assays confirmed that the cultivation of islets with EPO does not exert cytotoxic effects.

Cultivation of islets may induce hypoxic stress, which leads to elevated intracellular ROS levels [18, 19]. To assess whether EPO affects intracellular ROS levels of cultivated islets, we used islets from transgenic mice expressing mitochondrial or cytosolic H_2_O_2_ biosensors [12]. These biosensors change their fluorescence emission according to the ratio of oxidized (405 nm) to reduced roGFP2 (488 nm) depending on H_2_O_2_ levels. The quantitative analysis of the 405 nm / 488 nm ratio clearly demonstrated that EPO cultivation affects neither cytosolic nor mitochondrial H_2_O_2_ levels (Fig. 1d and e).

### Effect of EPO cultivation on the cellular composition and gene expression of isolated islets

**Fig. 2:**
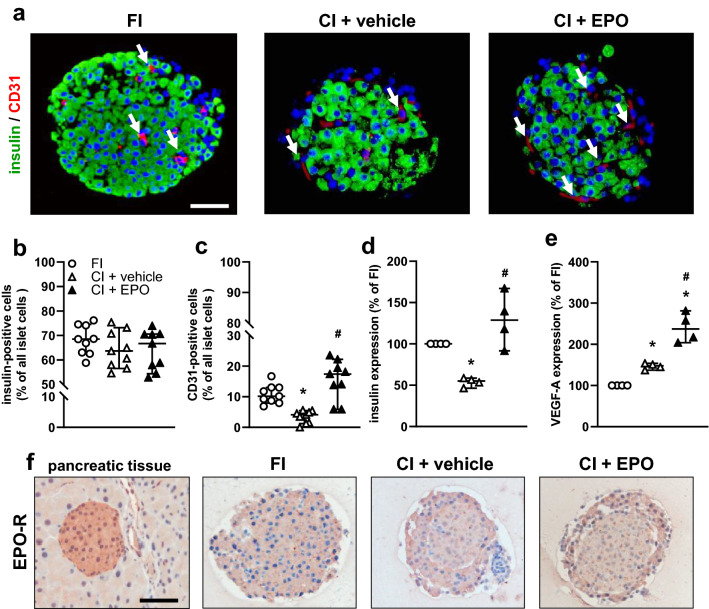
Effects of EPO cultivation on cellular composition and gene expression of isolated islets. **a** Representative immunofluorescence stainings of insulin/CD31 within freshly isolated islets (FI), vehicle-cultivated (CI + vehicle) and EPO-cultivated islets (CI + EPO). Cell nuclei are stained with Hoechst 33342 (blue). Scale bar: 25 µm. **b** and **c** Insulin-positive cells **b** and CD31-positive cells **c** of FI, CI + vehicle and CI + EPO in % of all islet cells (*n* = 9 each). Data are shown as scatter blots with median and 95% confidence interval. *P < 0.05 vs. FI, ^#^*P* < 0.05 vs. CI + vehicle. **d** and **e** Gene expression of insulin **d** and VEGF-A **e** of FI, CI + vehicle and CI + EPO (*n* = 4 each). FI were set 100%. Data are shown as scatter blots with median and 95% confidence interval. *P < 0.05 vs. FI, ^#^*P* < 0.05 vs. CI + vehicle. **f** Immunohistochemical stainings of EPOR in pancreatic tissue, FI, CI + vehicle and CI + EPO. Note: EPOR is expressed in islet cells. Scale bar: 45 µm

To investigate the effect of EPO on the cellular composition of isolated islets, we analyzed the number of insulin-producing cells and the number of CD31-positive cells by means of immunohistochemistry (Fig. 2a). Our quantitative analysis could not detect any differences in the number of insulin-positive cells in CI + EPO when compared to CI + vehicle and FI (Fig. 2b). However, a significantly lower fraction of CD31-positive cells was detected in CI + vehicle when compared to FI (Fig. 2a and c). In contrast, EPO exposure was capable of preventing the cultivation-induced reduction of CD31-positive cells (Fig. 2c).

Next, we examined the gene expression of insulin and VEGF-A by means of qRT-PCR. We found that the cultivation of islets reduces the insulin gene expression when compared to FI (Fig. 2d). Of note, this effect was completely abolished by the cultivation of the islets in the presence of EPO (Fig. 2d). The 24 h cultivation of islets increased VEGF-A gene expression compared to FI (Fig. 2e). This increase was further enhanced by EPO cultivation (Fig. 2e).

To exclude that the positive effects of EPO are mediated by an altered expression of EPOR after isolation or cultivation, we additionally analyzed the expression of this receptor in pancreatic tissue, FI, CI + vehicle and CI + EPO (Fig. 2f). The immunohistochemical analyses revealed that the expression of EPOR is neither affected by the isolation procedure nor by the cultivation.

### Effect of EPO cultivation on the revascularization of transplanted islets

**Fig. 3:**
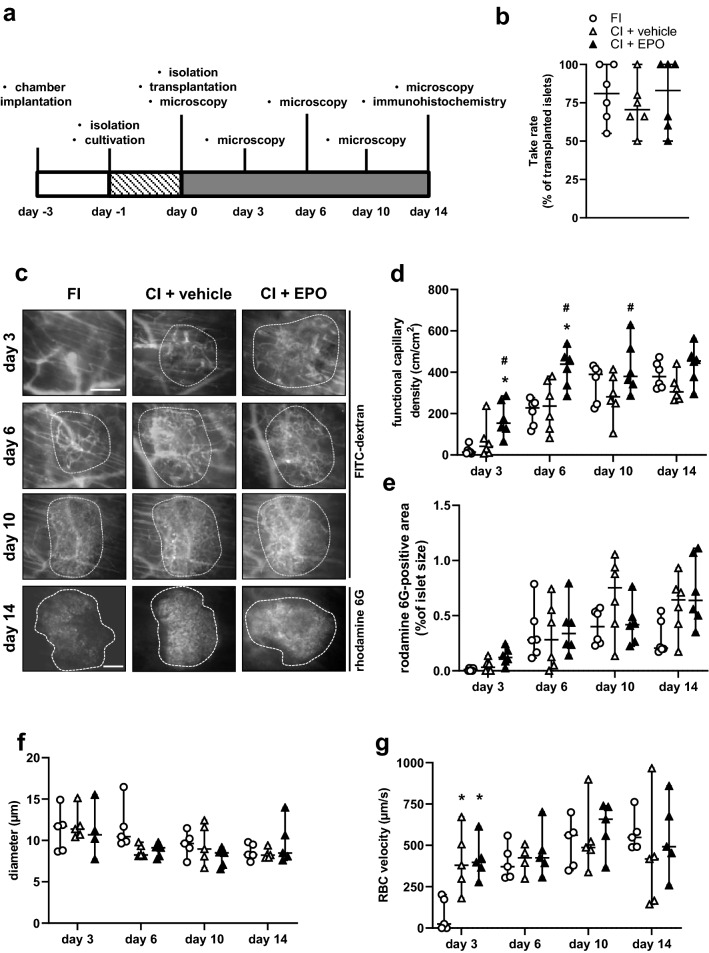
Effect of EPO cultivation on revascularization of transplanted islets. **a** Experimental protocol of islet transplantation: On day -3, dorsal skinfold chambers were implanted onto recipient mice. On day -1, islets were isolated and cultivated for 24 h in the presence of vehicle (CI + vehicle) or EPO (CI + EPO). On day 0, islets were isolated (FI) and FI, CI + vehicle and CI + EPO were transplanted onto the striated muscle of dorsal skinfold chambers. Subsequently, the grafts were analyzed by intravital fluorescence microscopy on days 0, 3, 6, 10 and 14. At the end of the experiments, the grafts were harvested for immunohistochemical analyses. **b** Take rate (%) of FI, CI + vehicle and CI + EPO on day 14 after transplantation into the dorsal skinfold chamber of FVB/N mice (*n* = 6 per group). Data are shown as scatter blots with median and 95% confidence interval. **c** Intravital fluorescence microscopic images of islets (marked by dotted lines) on days 3, 6, 10 and 14 after transplantation into dorsal skinfold chambers of FVB/N mice. The plasma marker FITC-dextran was used for the visualization of microvessels in blue light epi-illumination. Rhodamine 6G was injected to visualize the endocrine revascularization. Scale bar: 200 µm. **d** and **e** Functional capillary density (cm/cm^2^) **d** and rhodamine 6G positive area (% of islet size) **e** of FI, CI + vehicle and CI + EPO on days 3, 6, 10 and 14 after transplantation into the dorsal skinfold chambers of FVB/N mice (*n* = 6 per group). Data are shown as scatter blots with median and 95% confidence interval. *P < 0.05 vs.FI; ^#^*P* < 0.05 vs. CI + vehicle. **f** and **g** Diameter (µm) **f** and RBC velocity (µm/s) **g** of FI, CI + vehicle and CI + vehicle on days 3, 6, 10 and 14 after transplantation into the dorsal skinfold chambers of FVB/N mice (*n* = 5 per group). Data are shown as scatter blots with median and 95% confidence interval. *P < 0.05 vs.FI

FI islets, CI + vehicle and CI + EPO were transplanted onto the striated muscle tissue of dorsal skinfold chambers of recipient mice. The vascularization of the grafts was examined by means of intravital fluorescence microscopy on days 0, 3, 6, 10 and 14 (Fig. 3a). We found that cultivation of islets in the presence of EPO did not improve the take rate of the grafts, i.e., the fraction of engrafted islets on day 14 in relation to the total number of transplanted islets on day 0 (Fig. 3b). Repetitive intravital microscopy showed an increasing functional capillary density in the transplants of all groups throughout the entire observation period (Fig. 3c and d). Interestingly, we found a higher functional capillary density in particular on days 3 and 6 in CI + EPO animals when compared to CI + vehicle and FI controls. This clearly demonstrates that the cultivation of islets with EPO accelerates their revascularization.

Rhodamine 6G represents a marker for blood perfused endocrine tissue, because this fluorescent dye crosses the fenestrated endothelial lining and accumulates in the endocrine cells [20]. As expected, we measured an increased rhodamine 6G-positive area within all groups throughout the entire 14-day observation (Fig. 3c and e). On day 14, rhodamine 6G staining was higher in CI + EPO when compared to FI, however, this effect did not prove to be statistically significant (Fig. 3e). The measurement of additional microhemodynamic parameters showed that cultivation of islets with EPO did not significantly affect the intra-islet vessel diameter (Fig. 3f), whereas the cultivation procedure of islets itself had a positive effect on the RBC velocity during the initial period of transplantation on day 3 (Fig. 3g).

**Fig. 4:**
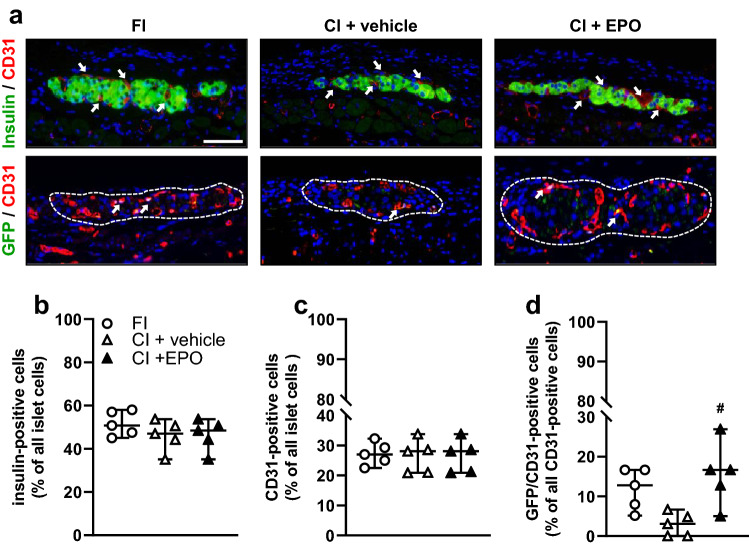
Immunohistochemical analysis of transplanted islets. **a** Immunofluorescence staining of insulin / CD31 (arrows = CD31-positive cells) and GFP / CD31 (arrows = GFP/CD31-positive cells) within FI, CI + vehicle and CI + EPO on day 14 after transplantation into the dorsal skinfold chamber of FVB/N mice. Scale bar: 25 µm. **b** and **c** Quantitative analysis of insulin- **b** and CD31-positive cells **c** within FI, CI + vehicle and CI + EPO on day 14 after transplantation into the dorsal skinfold chambers of FVB/N mice (% of all islet cells; *n* = 5 per group). Data are shown as scatter blots with median and 95% confidence interval. **d** Quantitative analysis of GFP/CD31-positive cells within transplanted islets on day 14 after transplantation into the dorsal skinfold chambers of FVB/N mice (in % of all CD31-positive cells) (*n* = 5 per group). Data are shown as scatter blots with median and 95% confidence interval. ^#^*P* < 0.05 vs. CI + vehicle

Finally, we analyzed the number of intra-islet blood vessels by means of immunohistochemistry on day 14 (Fig. 4a). We did not observe any differences in the number of insulin-positive cells and CD31-positive cells between FI, CI + vehicle and CI + EPO (Fig. 4b and c). Of interest, we detected a significantly increased number of GFP/CD31-positive cells in CI + EPO when compared to CI + vehicle. This indicates that EPO may protect intra-islet endothelial cells from stress-induced cell death during cultivation (Fig. 4a and d).

## Discussion

We have recently shown that 5-day pretreatment of recipient mice with EPO significantly accelerates the revascularization of transplanted islets [10]. However, in clinical practice there is little time between receiving the appropriate donor pancreas and the transplantation itself. Hence, pretreatment of patients with EPO is hard to realize in clinical practice [10]. In the present study, we analyzed whether cultivation of isolated islets with EPO prior to transplantation improves their posttransplant revascularization. Our results demonstrate that cultivation of islets with EPO does not affect their viability and cellular composition, and does not induce ROS formation. However, EPO cultivation preserves old blood vessels within the grafts during the initial phase after transplantation.

It is controversially discussed whether the cultivation of islets prior to transplantation leads to similar results compared to FI [21, 22]. Nonetheless, the cultivation has several advantages. For instance, it allows to improve viability by the cultivation of islets in the presence of anti-apoptotic compounds and allows the shipping of the islets to remote clinical transplant centers [23, 24]. Moreover, a brief cultivation for 24 h to 48 h prior to transplantation may allow recovery from cellular stress, induced by the islet isolation procedure and may allow the depletion of passenger leukocytes [25, 26].

EPO exerts anti-apoptotic and anti-inflammatory effects [27–29]. On the other hand, Zhao et al. [30] reported that the binding of EPO to EPOR is also capable of inducing ROS formation via activation of JAK2-STAT5 signaling. To exclude that subsequent effects are influenced by ROS-mediated changes in cell viability, we have first analyzed the effect of EPO cultivation on ROS formation and islet viability. In fact, EPO neither induced cell death nor affected the formation of ROS.

Further we studied the cellular composition of cultured and freshly isolated islets. We did not detect any differences in the fraction of insulin-positive cells between the groups. However, we found a significantly reduced insulin gene expression in CI + vehicle when compared to FI. Of note, this reduction of tissue gene expression was not detectable after cultivation of islets with EPO. This may be explained by the fact that pancreatic β-cells are positive for EPOR [7, 31] and EPO signaling activates JAK2-STAT5 pathways [32]. Because knockout of STAT5 dramatically reduces insulin secretion [33], EPO may increase insulin expression via STAT5 activation.

The analysis of the vascular fraction in FI, CI + vehicle and CI + EPO revealed that the cultivation procedure itself reduces the number of endothelial cells. This is in line with previous studies reporting a progressive loss of endothelial cells during islet cultivation [34, 35]. Of interest, EPO is capable of counteracting this endothelial cell loss during cultivation. Trincavelli et al. [36] investigated the effects of different erythropoietin derivatives such as EPOα, darbepoetin (DarbEPO) and continuous EPOR activator (CERA) on EPOR desensitization and signaling resensitization. They found that EPO increases endothelial cell viability through a mechanism involving STAT5 activation [36]. Therefore, it is tempting to speculate that EPO protects intra-islet endothelial cells against cultivation-induced cell death.

To investigate whether EPO cultivation improves the revascularization of transplanted islets, we implanted dorsal skinfold chambers onto recipient mice and subsequently transplanted FI, CI + vehicle and CI + EPO onto the striated skin muscle tissue. We detected an increased functional capillary density until day 10 after transplantation in all groups. Of note, this effect was most pronounced in the group of CI + EPO. It is well known that pro-angiogenic factors such as VEGF-A stimulate the formation of new blood vessels [37, 38]. VEGF-A is mainly produced by α- and β-cells [39] and regulates angiogenesis by inducing endothelial cell proliferation, migration and survival [40]. Choi et al. [9] detected increased VEGF mRNA levels in EPO-treated db/db mice when compared to controls. We herein detected a significantly increased VEGF-A expression in EPO-cultivated islets when compared to both controls. Therefore, we assume that the accelerated revascularization in the EPO-cultivated group is triggered by VEGF-A.

We did not detect significant differences in the take rate, blood vessel fenestration and vessel diameter between the three experimental groups. However, RBC velocity of CI + vehicle and CI + EPO was significantly increased on day 3 when compared to FI. This may support the revascularization process during the initial phase after transplantation by an increased nutrient and oxygen supply.

Finally, we assessed the cellular composition of the grafts 14 days after transplantation. As expected, we observed no differences in the ratio of insulin- and CD31-positive islet cells between the study groups. To analyze the origin of intra-islet endothelial cells, we used islets from Tie2/GFP-positive donor mice. Our data show that donor islet endothelial cells contributed to ~ 10% to the islet vasculature in the group of FI. This is in line with previous studies demonstrating that the revascularization of transplanted islets is mainly caused by recipient endothelial cells of host origin [41–43]. In CI, we measured a decreased number of intra-islet endothelial cells. Of note, the cultivation of islets with EPO was capable of preventing this endothelial cell loss. This may be explained by our in vitro observation, which shows that cultivation of islets in the presence of EPO results in a higher number of intra-islet endothelial cells. Hence, the increased number of recipient microvessels in EPO-pretreated islets may contribute to the accelerated graft revascularization through inosculation with the host vasculature.

Clinical islet transplantation is usually performed by an intraportal infusion of islets [44]. Recently, Chen et al. [45] visualized for the first time the revascularization of intraportally transplanted islets in diabetic mice by panoramic and in-depth imaging of the graft microstructure. This approach revealed that although the transplanted islets have access to nutrients and oxygen immediately after transplantation, an accelerated graft-hepatic integration by blood microvessels plays an essential role for long-term graft survival. In the present study, we did not transplant islets intraportally nor did we use diabetic mice as recipients. However, the herein observed beneficial effects of EPO exposure of isolated islets may have impact on clinical islet transplantation by improving the revascularization of intraportally transplanted islets [46].

Taken together, we herein demonstrate that cultivation of islets with EPO significantly accelerates the revascularization of transplanted islets during the initial days after transplantation. This is most probably due to the enhanced gene expression of insulin and VEGF-A driven the inosculation. Besides EPO, darbepoietin (DPO)-α, a long lasting analogue of EPO has also been reported to stimulate angiogenesis in vitro and in vivo [47, 48]. Moreover, we have shown that treatment of mice with DPO after islet transplantation increases the blood volume flow in the grafts [49]. Therefore, cultivation of islets with EPO or even DPO prior to transplantation may represent a successful strategy to improve the outcome of clinical islet transplantation.
